# Pulse Waveform Analysis of the Ocular Blood Flow Using Laser Speckle Flowgraphy before and after Glaucoma Treatment

**DOI:** 10.1155/2019/1980493

**Published:** 2019-10-03

**Authors:** Satoko Masai, Kyoko Ishida, Ayako Anraku, Tetsuro Takumi, Goji Tomita

**Affiliations:** Department of Ophthalmology, Toho University Ohashi Medical Center, 2-22-36, Ohashi Meguro-ku, Tokyo 153-8515, Japan

## Abstract

Although reduction in intraocular pressure (IOP) is the principle of glaucoma treatment, impaired ocular blood flow is believed to play a role in the progression of glaucoma. This study evaluated the effect of glaucoma treatment on pulse waveforms for optic nerve head (ONH) microcirculation in patients with glaucoma. Fifty-one subjects were included on the basis of the glaucoma treatment administered, which involved instillation of prostaglandin (PG) analogs (PG group; *n* = 28) or trabeculectomy (trabeculectomy group; *n* = 23). ONH blood flow, represented by the mean blur rate (MBR_T_) and pulse waveforms, was measured using laser speckle flowgraphy before and 1 and 3 months after treatment. Three months after treatment, IOP exhibited a significant decrease (*p* < 0.05). Although there was no significant change in MBR_T_ after treatment, the acceleration time index (ATI) significantly decreased (*p*=0.034) in the PG group. In the trabeculectomy group, there was no significant change in the MBR_T_ after treatment, while fluctuation (*p*=0.019) and blowout score (BOS) (*p*=0.036) exhibited significant decrease and increase, respectively. Multiple regression analysis showed that mean deviation was significantly associated with the rate of change in the BOS (*p*=0.013), age was a significant contributing factor for the rate of change in fluctuation in the trabeculectomy group, reflection was significantly associated with the ATI (*p*=0.037) in the in the PG group. Both glaucoma treatments can change the pulse waveforms, with MBR_T_ remaining unchanged, and IOP reduction owing to the treatment may contribute to stable blood flow in the tissue area of the ONH. As impaired ocular blood flow plays a role in the progression of glaucomatous damage, it would be beneficial if glaucoma treatment could improve the stability of ONH microcirculation.

## 1. Introduction

Reduction in intraocular pressure (IOP) is the principle of glaucoma treatment, and many clinical trials have proven that decrease in IOP prevents deterioration of visual function [1–4]. However, some patients show progression of visual-field defects despite a decrease in IOP. It is believed that other risk factors, such as impaired ocular blood flow, play a role in the progression of glaucomatous damage [5–7]. Therefore, both decrease in IOP and improvement in optic nerve head (ONH) microcirculation have important clinical implications.

Prostaglandin (PG) analogs are used as the first-line treatment for glaucoma because of their strong ability to lower IOP and ease of application once a day. Some studies have reported that PG analogs lower IOP and increase ocular blood flow [8–11]. A previous study used an isometric-tension recording system to show that PG analogs prevented endothelin-1-induced contraction in rabbit ciliary arteries; moreover, improvement in ocular blood flow was attributed to relaxation of the ocular vessels [12].

Trabeculectomy is commonly performed for patients with glaucoma whose IOP cannot be controlled by medical therapy. The effect of trabeculectomy on ocular blood flow remains unclear. Some studies have reported an increase in ocular blood flow after trabeculectomy, whereas some found no such increase [13–16]. While the reasons for this discrepancy in findings are not clear, different techniques for measuring the blood flow, different sample sizes, different groups of subjects, and other confounding factors such as the use of ocular hypotensive agents could be responsible.

Laser speckle flowgraphy (LSFG) utilizes the laser speckle phenomenon for the measurement of ocular blood flow in a noninvasive manner [17]. LSFG provides the mean blur rate (MBR), which is proportional to blood velocity and has been used for the measurement of relative differences in blood flow in the optic nerve head (ONH) [18–20]. Furthermore, LSFG enables the recording of changes in pulse waveforms for the MBR, which are synchronized with the cardiac cycle [21–24]. Shiga et al. reported that pulse waveform parameters such as skew and the acceleration time index (ATI) could differentiate healthy eyes from those with mild normal-tension glaucoma (NTG) [25]. Another study showed that the skew and blowout time (BOT) correlated with the mean deviation (MD) in patients with primary open-angle glaucoma (POAG) [26]. However, to the best of our knowledge, only one trabeculectomy study [27] has performed pulse waveform analysis of ocular blood flow before and after glaucoma treatment. The purpose of the present study was to evaluate the effect of PG analog instillation or trabeculectomy on the pulse waveforms for ONH microcirculation using LSFG.

## 2. Materials and Methods

### 2.1. Study Subjects

This prospective, observational study was approved by the institutional review board of Toho University Ohashi Medical Center (approval number 12-83) and adhered to the tenets of the Declaration of Helsinki. Subjects were recruited from the Department of Ophthalmology outpatient clinic at Toho University medical center between January 2013 and April 2017. According to the guidelines, we obtained written informed consent from all patients for a prospective study. The inclusion criteria were as follows: a clinical diagnosis of POAG or NTG by slit lamp with angle examinations, IOP measurements, fundus photography, optical coherence tomography with disc and macular ganglion cell analysis, and reliable visual-field tests, a best-corrected visual acuity of at least 20/40, and a refractive spherical equivalent (SE) ranging between −8.0 diopters (D) and +3.0 D. For the PG analogs group, patients without glaucoma medications were recruited. For the trabeculectomy group, patients who were scheduled for trabeculectomy were included regardless of glaucoma medications.

The exclusion criteria were as follows: intraocular eye disease (other than POAG or NTG), significant cataract that could influence refractive errors and visual fields, systemic disease including diabetes mellitus known to affect the visual field, and poor image quality and unreliable measurement of LSFG. Patients with hypertension were included. However, the subjects were excluded from the analysis if changes in systemic medications were observed during the follow-up period. Only one eye from each subject was randomly included in this study.

In the PG analogs group, patients randomly received 0.005% latanoprost or 0.0015% tafluprost as the first-line treatment for glaucoma.

In the trabeculectomy group, patients continued using their ocular hypotensive medications up to the time of surgery, which was performed when there was one or both of the following indications despite use of maximum tolerable medications: documented and confirmed visual field and/or optic disc progression and an IOP that was clinically too high for the level of glaucomatous damage. Patients were scheduled for glaucoma surgery in the university hospital. Laser suture lysis and bleb needling revision were performed as clinically indicated within 2 months after surgery at the discretion of the treating surgeon.

### 2.2. Laser Speckle Flowgraphy

**Figure 1:**
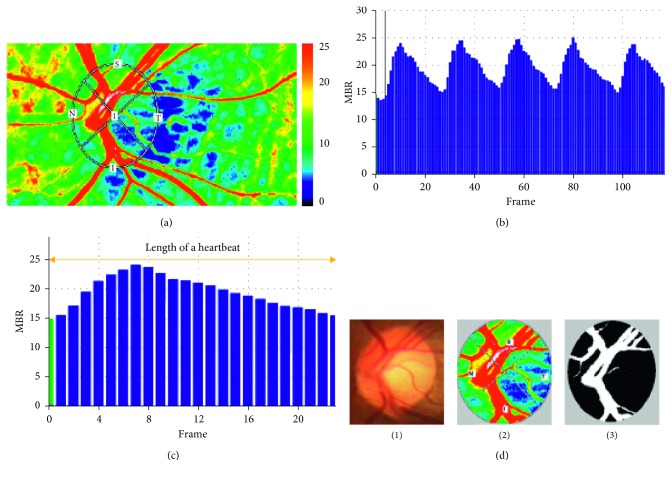
Analysis of pulse waveforms for the optic nerve head (ONH) using laser speckle flowgraphy (LSFG). (a) Representative color-coded composite map. By this circle rubber band at the ONH, the mean blur rate (MBR) and other waveform parameters can be measured. (b) Pulse wave showing changes in the MBR, which is tuned to the cardiac cycle for 4 s (the total number of frames is 118 in a scan). (c) The change in the MBR in one heartbeat. (d) (1) A fundus photograph showing the ONH. (2) The MBR for ONH is automatically calculated. (3) Vessel extraction function automatically detected vessel and tissue areas within the ONH. The black area represents the tissue area, while the white area represents the vessel area.

ONH blood flow was evaluated using LSFG (LSFG-NAVI version 3.1.39.2 software, Softcare Ltd., Fukuoka, Japan). The principle and methods of LSFG have been described in previous studies [17, 28]. Briefly, the instrument comprises a fundus camera equipped with a diode laser (wavelength, 830 nm) as the light source and a digital charge-coupled device camera (resolution, 750 × 360 pixels) as the detector. When a rough surface is illuminated with a laser, the background light gives the appearance of a consistent scatter pattern, i.e., the speckle pattern. Moving erythrocytes in the blood vessels cause distinct variations in the speckle pattern. After acquisition of the speckle pattern produced by the erythrocytes with a digital camera, the information can be analyzed by the embedded analysis software in order to generate flow information. The primary output parameter of LSFG is the MBR, representing the relative blood flow velocity and is expressed in arbitrary units (AUs). The ONH margins were measured with an ellipsoidal band, and the position of the ONH was saved in the system software (Figure 1). The MBR images of the ONH were recorded at a rate of 30 frames/s over a 4 s period and automatically detected the beginning and the end of the heartbeat recorded within 4 s. The total number of frames was 118 (Figure 1(b)). Images corresponding to the identical phases within 1 heartbeat duration were synthesized to one image sequence [27]. The averaged MBR for a heartbeat was calculated and displayed as a heartbeat map (Figure 1(c)).

The LSFG analysis software “vessel extraction function” automatically detected vessel and tissue areas within the ONH and calculated the mean MBR in all areas of the optic disc, the vessel area of the optic disc, and the tissue area of the optic disc (MBR_T_) (Figure 1(d)). We used MBR_T_ for our analysis because it has been shown to be strongly correlated with absolute blood flow values measured by the microsphere method or the hydrogen gas clearance method in primates and rabbits [29–31]. MBR_T_ appears to reflect the blood flow in the retrolaminar region, which is served primarily by the short posterior ciliary circulation [31].

**Figure 2:**
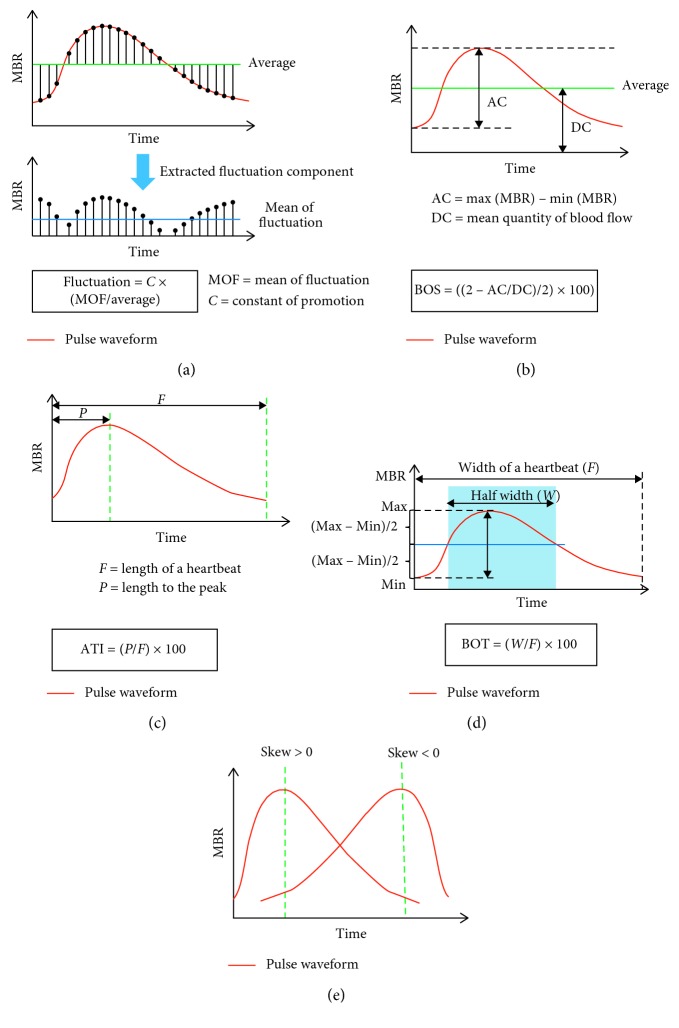
Definition and calculation of pulse waveform parameters. (a) Fluctuation is a parameter indicating instability of the blood flow. It is proportional to the mean fluctuation in the mean blur rate (MBR) divided by the average MBR in a waveform. Fluctuation = constant of promotion × mean of fluctuation/MBRaverage. (b) The blowout score (BOS) indicates the amount of the blood flow volume in one heartbeat and is calculated from the difference of the maximum and minimum MBR as well as the average waveform distribution. BOS = 100 × {2 − (MBRmax − MBRmin)/MBRavergae}/2. A high BOS is an indicator of high constancy of blood flow during the cardiac cycle. (c) The acceleration time index (ATI) is defined as the ratio of the time before the pulse wave peak value is reached in a heartbeat. ATI = 100 × length to peak/length of a heartbeat. A smaller ATI indicates a more rapid increase in the MBR to the peak. (d) Blowout time (BOT) is defined as the ratio of the half width (i.e., the time that the waveform is higher than half of the mean of the minimum and maximum signals) in a heartbeat. BOT = 100 × half width/width of a heartbeat. A higher BOT indicates that a high level of MBR is maintained for a larger proportion of a single heartbeat. (e) Skew represents asymmetry of the MBR waveform in the waveform distribution. If the waveform is completely symmetrical, the skew is zero. When the peak of the pulse wave comes faster than that of the symmetrical waveform, the skew increases, and when the peak comes slower, the skew decreases.

In addition, the LSFG software provides numerous parameters characterizing the shape of the MBR waveform during one cardiac cycle (pulse waveform analyses) for assessment of the dynamics of ocular blood flow. The definitions and calculations of the pulse waveform parameters have been described in detail in previous studies [21, 22, 32, 33]. In the present study, five pulse waveform parameters were used for analysis (Figure 2): (1) Fluctuation is a parameter indicating the instability of the blood flow. It is proportional to the mean fluctuation in the MBR divided by the average MBR in a waveform. Fluctuation = constant of promotion × mean of fluctuation/MBRaverage. (2) The blowout score (BOS) indicates the amount of the blood flow volume in one heartbeat and is calculated from the difference of the maximum and minimum MBR as well as the average waveform distribution. BOS = 100 × {2 − (MBRmax − MBRmin)/MBRaverage}/2. A high BOS is an indicator of high constancy of blood flow during the cardiac cycle. (3) The acceleration time index (ATI) is defined as the ratio of the time before the pulse wave peak value is reached in a heartbeat. ATI = 100 × length to peak/length of a heartbeat. A smaller ATI indicates a more rapid increase in the MBR to the peak. (4) Blowout time (BOT) is defined as the ratio of the half width (i.e., the time that the waveform is higher than half of the mean of the minimum and maximum signals) in a heartbeat. BOT = 100 × half width/width of a heartbeat. A higher BOT indicates that a high level of MBR is maintained for a larger proportion of a single heartbeat. (5) Skew represents the asymmetry of the MBR waveform in the waveform distribution. If the waveform is completely symmetrical, the skew is zero. When the peak of the pulse wave comes faster than that of the symmetrical waveform, the skew increases, and when the peak comes slower, the skew decreases.

LSFG measurements were performed before and 1 and 3 months after treatment (instillation of PG analogs or trabeculectomy) between 13 : 00 pm and 15 : 00 pm. The pupils of the enrolled eyes were dilated using 0.4% tropicamide before the LSFG examination, and three consecutive measurements were obtained at each time point. The average of the three measurements was used for analysis.

### 2.3. Measurements of Clinical Parameters

The IOP was measured using a Goldmann applanation tonometer, and the value recorded on the day of the LSFG measurements was used for analysis. The systolic blood pressure (SBP), diastolic blood pressure (DBP), and heart rate (HR) were measured before LSFG. The mean blood pressure (MBP) and mean ocular perfusion pressure (MOPP) were calculated as follows:

(1) $$\begin{array}{c} \text{MBP}=\text{DBP}+\frac{1}{3}\left(\text{SBP}-\text{DBP}\right),\\ \text{MOPP}=\frac{2}{3}\text{MBP}-\text{IOP.} \end{array}$$

### 2.4. Visual-Field Analyses

Standard automated perimetry was performed using a Humphrey Field Analyzer (Carl Zeiss Meditec Inc., Dublin, CA, USA) with the 30-2 Swedish Interactive Threshold Algorithm. A glaucomatous visual-field change was defined by the presence of three or more significant (*p* < 0.05) nonedge-contiguous points, with at least one point at the *p* < 0.01 level in the pattern deviation plot, along with grading outside the normal limits in the glaucoma hemifield test. Visual-field tests were performed three times at diagnosis and considered reliable when fixation losses were <20%, false positives were <15%, and false negatives were <25%. The average MD of the two latest visual-field tests before treatment (instillation of PG analogs or trabeculectomy) was calculated.

### 2.5. Statistical Analysis

To investigate the impact of IOP decrease on ocular hemodynamics, data of eyes with ≥20% decrease in IOP at 3 months after treatment were included in the statistical analysis. The Wilcoxon signed-rank test was used to evaluate differences in values before and after treatment, while the Mann–Whitney *U* test was used to compare factors between the two groups. Categorical data were compared using chi-square tests. Stepwise multiple regression analyses were performed to determine factors contributing to the rate of change in the pulse waveform parameters. The data are reported as means ± standard deviations. A *p* value of <0.05 was considered statistically significant.

## 3. Results

In total, 51 subjects, including 28 in the PG analogs group and 23 in the trabeculectomy group, were recruited for this study. The demographic and ocular characteristics of the two groups are shown in Table 1. The values for age were 50.6 ± 10.8 years in the PG analogs group and 60.0 ± 9.7 years in the trabeculectomy group. The pretreatment IOP and the IOP decrease rate at 3 months were 16.0 ± 2.8 mmHg and 26.0% in the PG analogs group and 19.1 ± 3.8 mmHg and 42.2% in the trabeculectomy group, respectively. None of the subjects in the PG analogs group had received any pretreatment glaucoma medication. In contrast, all subjects in the trabeculectomy group had received topical glaucoma medications.

**Figure 3:**
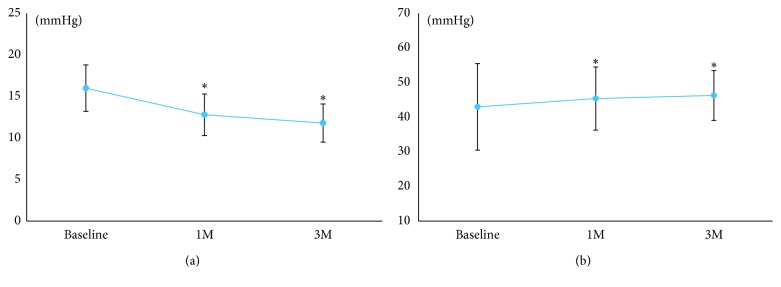
Changes in interocular pressure (IOP) and mean ocular perfusion pressure (MOPP) after the instillation of topical prostaglandin analogs. (a) IOP. (b) MOPP. Significant changes compared to the baseline are indicated with an asterisk (*p* < 0.05).

In the PG analogs group, there were significant decreases in IOP (Figure 3(a)) and increases in MOPP (Figure 3(b)) at 1 and 3 months after the instillation of the PG analogs (*p* < 0.05).

**Figure 4:**
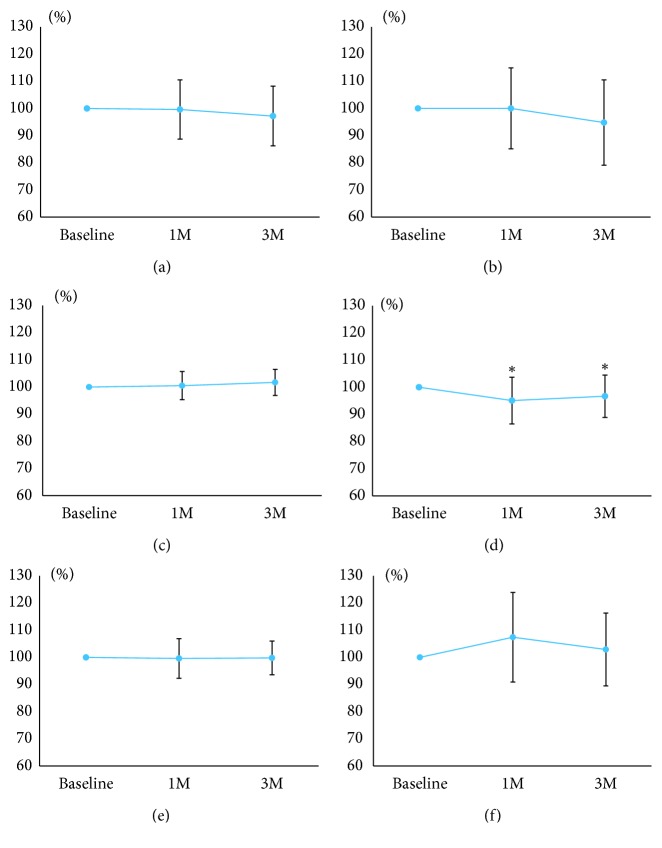
Changes in the mean blur rate (MBR) and pulse waveform parameters after the instillation of topical prostaglandin analogs. (a) The MBR in the tissue is of the optic disc. (b) Fluctuation. (c) Blowout score. (d) Acceleration time index. (e) Blowout time. (f) Skew. Significant changes compared to the baseline are indicated with an asterisk (*p* < 0.05).

Figure 4 shows the changes in MBR_T_ and pulse waveform parameters 1 and 3 months after the instillation of the topical PG analogs. There were no significant changes in MBR_T_ (Figure 4(a)), fluctuation (Figure 4(b)), BOS (Figure 4(c)), BOT (Figure 4(e)), and skew (Figure 4(f)). Significant changes compared to the baseline were observed in the ATI after treatment (*p* < 0.05) (Figure 4(d)).

**Figure 5:**
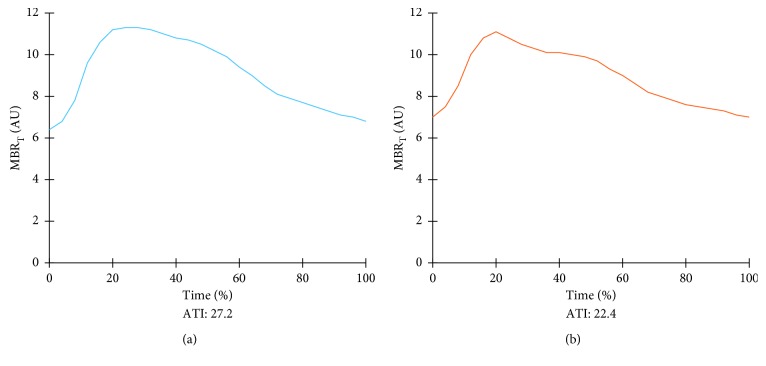
Pulse waveform analysis of ocular blood flow after prostaglandin analog treatment for glaucoma in a representative case. (a) The pulse waveform before treatment. (b) The acceleration time index (ATI) has decreased at 3 months after the instillation of topical prostaglandin analogs.

Table 2 shows the changes in ocular and systemic parameters at 3 months after the instillation of PG analogs. After treatment, IOP exhibited a significant decrease (*p* < 0.001), while MOPP exhibited a significant increase (*p*=0.004). There was no significant change in MBR_T_ after treatment. However, the ATI significantly decreased after treatment (*p*=0.034). A representative case from the PG analogs group is shown in Figure 5. A smaller ATI indicates a more rapid increase in the MBR to the peak. Multiple regression analysis for the 28 subjects in the PG analogs group was performed with the rate of change in the ATI at 3 months as the dependent variable, and age, sex, history of hypertension, refraction, MD, pretreatment MBR_T_, and rate of IOP decrease at 3 months as explanatory variables. The findings revealed that refraction (slope, 0.980; *β* = 0.397, 95% confidence interval (CI), 0.066–1.894; *p*=0.037) was a significant contributing factor for the rate of change in the ATI at 3 months.

**Figure 6:**
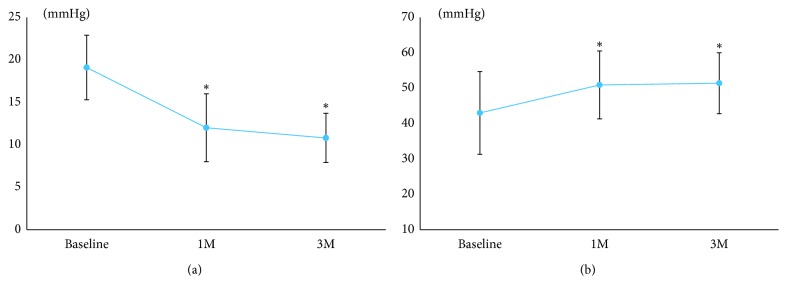
Changes in interocular pressure (IOP) and mean ocular perfusion pressure (MOPP) after trabeculectomy. (a) IOP. (b) MOPP. Significant changes compared to the baseline are indicated with an asterisk (*p* < 0.05).

In the trabeculectomy group, there were significant decreases in IOP (Figure 6(a)) and increases in MOPP (Figure 6(b)) at 1 and 3 months after trabeculectomy (*p* < 0.05).

**Figure 7:**
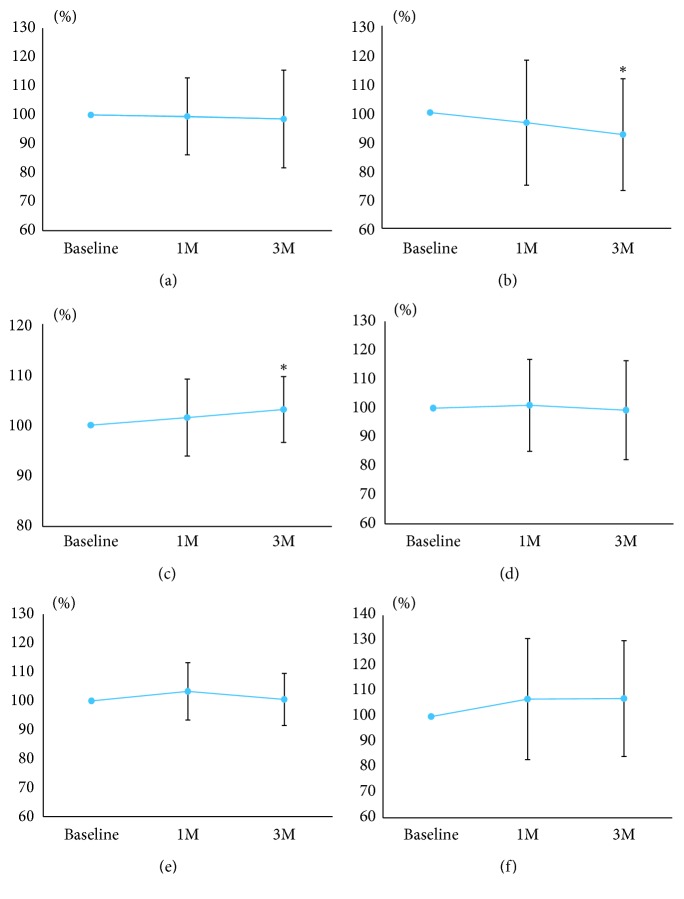
Changes in the mean blur rate (MBR) and pulse waveform parameters after trabeculectomy. (a) The MBR in the tissue is of the optic disc. (b) Fluctuation. (c) Blowout score. (d) Acceleration time index. (e) Blowout time. (f) Skew. Significant changes compared to the baseline are indicated with an asterisk (*p* < 0.05).

Figure 7 shows the changes in MBR_T_ and pulse waveform parameters 1 and 3 months after trabeculectomy. There were no significant changes in MBR_T_ (Figure 7(a)), the ATI (Figure 7(d)), BOT (Figure 7(e)), and skew (Figure 7(f)) after trabeculectomy. Significant changes compared to the baseline were observed in fluctuation (Figure 7(b)) and the BOS (Figure 7(c)) 3 months after trabeculectomy (*p* < 0.05).

**Figure 8:**
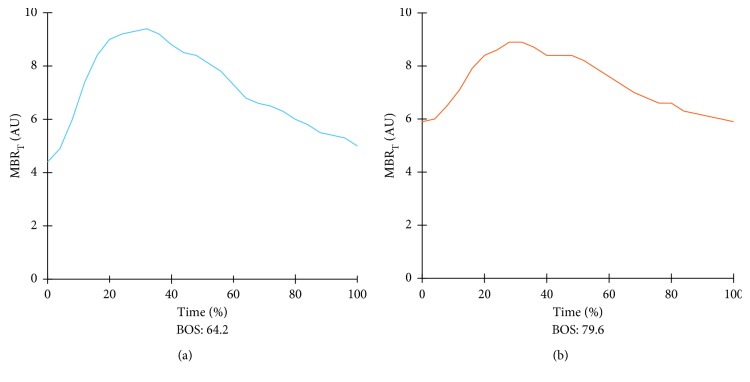
Pulse waveform analysis of ocular blood flow after trabeculectomy for glaucoma in a representative case. (a) The pulse waveform before treatment. (b) The blowout score (BOS) has increased at 3 months after trabeculectomy.

Table 3 shows the changes in ocular and systemic parameters at 3 months after trabeculectomy. After trabeculectomy, IOP exhibited significant decrease (*p* < 0.001), while MOPP exhibited significant increase (*p*=0.002). Although there was no significant change in MBR_T_ after treatment, fluctuation (*p*=0.019) and the BOS (*p*=0.036) exhibited significant decrease and increase, respectively. A representative case from the trabeculectomy group is shown in Figure 8. The average waveform of the MBR flattened after trabeculectomy. Multiple regression analysis for the 23 subjects in the trabeculectomy group was performed with the rate of change in the BOS or fluctuation at 3 months as the dependent variable, and age, sex, history of hypertension, refraction, MD, pretreatment MBR_T_, rate of IOP decrease at 3 months, and number of topical medications before trabeculectomy as explanatory variables. The baseline MD (slope, 0.446; *β* = 0.511; 95% CI, 0.106–0.787; *p*=0.013) was a significant contributing factor for the rate of change in the BOS at 3 months, and age (slope, 1.044; *β* = 0.526; 95% CI, 0.278–1.809; *p*=0.010) was significantly related to the rate of change in fluctuation at 3 months.

## 4. Discussion

The present study revealed that the pulse waveforms for ONH microcirculation can be changed to a more stable flow by glaucoma treatment, although there was no significant increase in the quantity of microcirculation (MBR_T_) after treatment.

Some studies have shown that PG analogs had vasodilatory effects in animal experiments [12, 34, 35]. In healthy subjects, Tamaki et al. reported that tissue-blood velocity in the ONH increased after a single instillation of 0.005% latanoprost [36]. This result was confirmed by Gherghel et al., who demonstrated that ocular perfusion in the ONH, measured by the Heidelberg retina flowmeter (HRF), improved after 3 months and 6 months of treatment with 0.005% latanoprost in treatment-naïve patients with POAG [8]. In addition, Mayama et al. reported that topical instillation of 0.0015% tafluprost increased the ONH microcirculation in normal and laser-induced glaucomatous eyes of monkeys [37]. This result is consistent with the findings in a study showing that topical tafluprost increased the MBR in the ONH of patients with glaucoma and a myopic disc [9]. These results suggest that PG analogs may not only lower IOP but also improve ocular microcirculation. In the current study, the ATI significantly decreased after treatment with PG analogs. A previous study found that the ATI in patients with NTG was significantly higher than that in subjects with healthy eyes. It is speculated that the increase in the ATI may be due to endothelial dysfunction or increased vascular resistance [25]. Therefore, our result implies that the instillation of topical PG analogs may lower vascular resistance. This implication is compatible with the result of an experimental study showing that PG analogs prevented endothelin-1-induced contraction in rabbit ciliary arteries [12]. However, in the present study, there was no significant increase in ONH microcirculation measured as MBR_T_ after treatment. We believe that the effect of PG analogs on ocular blood flow may be limited. Some other studies reported that there was no significant change in ocular circulation after the instillation of latanoprost [38–40]. Akaishi et al. reported that the increase in the ONH blood flow induced by tafluprost was greater than that induced by latanoprost [11]. In the present study, 12 and 16 subjects received 0.005% latanoprost and 0.0015% tafluprost, respectively. There was no significant difference between the two groups with regard to the rate of change in MBR_T_ and the ATI (data not shown).

With regard to the effect of trabeculectomy on ocular blood flow, previous results are contradictory. Our results are consistent with those of studies that found no significant change in ONH microcirculation evaluated by LSFG or HRF after trabeculectomy [15, 16, 27]. In contrast, Berisha et al. reported that ONH microcirculation measured by scanning laser Doppler flowmetry (SLDF) significantly increased after trabeculectomy, with a significant association between the increase in MOPP and the increase in ONH microcirculation [14]. Discrepancy between the results published by Berisha et al. and ours may be caused by the measurement depth. LSFG reflects ONH blood flow in the retrolaminar region, which is primarily supplied by short posterior ciliary arteries, while SLDF mainly measures the microcirculation on the anterior portion of the ONH, which is supplied by the central retinal artery [30]. Moreover, three other studies evaluating the effects of trabeculectomy on pulsate ocular blood flow (POBF) showed that trabeculectomy resulted in significant decrease in IOP and increase in ocular blood flow [13, 41, 42]. Recent studies using enhanced-depth imaging spectral-domain optical coherence tomography revealed that IOP decrease after trabeculectomy caused choroidal thickening and that the increased choroidal thickness was associated with an increase in the intravascular and extravascular compartments in the choroid [43, 44]. Because POBF is primarily determined by the choroidal circulation [41, 42], increase in POBF after trabeculectomy is justified. In the present study, there was a significant decrease in IOP and increase in MOPP after trabeculectomy; however, there was no significant change in the quantity of ONH microcirculation (MBR_T_). We speculated that ocular hemodynamic autoregulation may have played a role. The vascular bed in the ONH is considered to exhibit autoregulation, which is the ability of the vascular bed to maintain its blood flow despite changes in the perfusion pressure [45, 46]. Our results suggest that an increase in MOPP may have limited influence on the degree of ONH microcirculation in patients with glaucoma.

In the present study, however, two pulse waveform parameters, fluctuation and the BOS, exhibited significant changes after trabeculectomy. Takeshima et al. [27] reported waveform changes in ONH blood flow by using LSFG in 48 patients with glaucoma who had undergone trabeculectomy in 2019. Our study is the second to report on the same factor. In their study, similar to ours, MBR_T_ remained unchanged after trabeculectomy; however, the postoperative BOS increased and the resistivity index (RI) decreased. The strong inverse relationship between the BOS and RI is an expected result considering the formula for calculating the BOS and RI. They also found no change in skew, ATI, and BOT after trabeculectomy. Fluctuation in ONH blood flow after trabeculectomy was only measured by us and it indicates the instability of the blood flow [33]. Both diurnal and nocturnal fluctuation in IOP decreased after trabeculectomy [47, 48]. Our result indicates that trabeculectomy decreases the fluctuation in the blood flow as well as IOP at least when measured during the daytime. The BOS represents the constancy of the blood flow in each heartbeat. It is considered to be an index of blood vessel resistance and shows a higher value when the blood flow is evaluated as stable. A previous study reported that the BOS was a significant contributing factor for the severity of carotid atherosclerosis [49], while another showed that the BOS significantly decreased with age [21]. In the present study and the study by Takeshima et al., MBR remained unchanged, the BOS significantly increased, and fluctuation significantly decreased after trabeculectomy. These observations suggest that the decreased IOP after trabeculectomy changes the hemodynamics to yield more stable perfusion during a single heartbeat in the tissue region of the ONH.

Multiple regression analysis showed that baseline MD was a significant contributing factor for the rate of change in the BOS, and age was significantly related to the rate of change in fluctuation at 3 months in the trabeculectomy group. Takeshima et al. [27] concluded that younger age, worse baseline MD, and large IOP reduction increase are significantly associated with postoperative BOS increase. Age has a significant negative association with the BOS in normal subjects, suggesting an effect on less elastic vessel walls owing to atherosclerosis [27]. According to our and Takeshima's findings, the beneficial effects of trabeculectomy on hemodynamics are less likely in elderly patients.

In contrast to the study by Takeshima et al., better baseline MD was significantly associated with BOS increase after trabeculectomy in the current study. Average baseline MD in the trabeculectomy group was better (−14.3 dB) in the current study than in the study by Takeshima et al. (−19.7 dB). Other previous studies have not discussed the association of hemodynamic change and the severity of baseline VF. Further research is needed to elucidate whether the change in hemodynamics after trabeculectomy is associated with VF severity and furthermore, whether improved hemodynamics result in better prognosis in glaucoma.

A large reduction in IOP was significantly associated with a postoperative increase in the BOS in the study conducted by Takeshima et al. [27]. In current glaucoma practice, trabeculectomy is the gold standard for the surgical treatment of patients with advanced glaucoma. To investigate the impact of IOP decrease on ocular hemodynamics, eyes with ≥20% decrease in IOP at 3 months after treatment were included in the current study, and the actual IOP reduction was 42.2% after trabeculectomy. After large IOP reduction, postoperative increase in the BOS and decrease in fluctuation were observed in the current study. Histologic studies have demonstrated that compression and posterior deflection of the lamina cribrosa (LC) may cause damage to axons and blockage of axon flow [50]. However, medical or surgical IOP reduction causes anterior displacement of the LC in most glaucomatous eyes [51–54] and a larger IOP reduction is related to a larger LC depth shallowing [51]. Our study and the study by Takeshima et al. indicate that IOP reduction may be related to the flattening of the MBR waveform with a decrease in fluctuation and an increase in the BOS. Eyes with sustained reduction of LC depth over a long period have a slow rate of progressive retinal nerve fiber thinning after trabeculectomy [53]. Further studies are necessary to investigate the association between structural changes and MBR waveform changes in ONH after IOP reduction in glaucoma.

Refraction was significantly associated with the rate of change in the ATI at 3 months in the PG treatment group. Eyes with greater myopia showed a tendency for lower ATI after treatment. The sclera of eyes with myopia is thinner than that of eyes without myopia [55]. A decrease in IOP leads to a consequent decrease in axial length [43, 56] and to morphological changes in the posterior pole and/or peripapillary sclera, including shrinkage of the peripapillary sclera, decrease in the LC depth, flattening of the peripapillary scleral insertion into the optic disc, and decrease in the angle of the scleral protrusion temporal to the optic disc [57]. These biomechanical properties related to refraction may influence the change in blood flow in ONH after IOP reduction.

Our study has several limitations. First, we did not evaluate diurnal variations in ONH microcirculation. Because the ONH microcirculation measured by LSFG exhibited a significant diurnal fluctuation [32, 58], it may have influenced our results. However, the 1- and 3-month IOP, LSFG, BP, and HR measurements and the corresponding baseline measurements were performed at almost the same time. Second, regarding the hemodynamic study, there is a possibility that a systemic condition may have affected the ocular hemodynamics measured by LSFG. During the study period, we measured blood pressure, heart rate, mean ocular perfusion pressure, and interviewed patients and confirmed that there were no significant changes in these values and no change in systemic and topical medications during the study period. We did not exclude patients with hypertension, which may cause insufficiency in the ocular blood flow; this may have affected our results. However, when we analyzed factors related to the rate of change in pulse waveform parameters, the history of hypertension was not a significant contributing factor. Third, all subjects in the trabeculectomy group discontinued all antiglaucoma medications after surgery. Because topical antiglaucoma medications may affect ocular blood flow, this could have influenced our results. However, numbers of topical medication before trabeculectomy did not exhibit significance in the multiple regression analyses. Takeshima et al. also concluded that no drugs showed a significant association with postoperative changes in the BOS [27]. Furthermore, Tamaki et al. [15] reported that there was no increase in ocular blood flow after trabeculectomy. They also measured ONH blood flow before and after the needling procedure with no change in topical glaucoma medication and showed that ONH blood flow did not change significantly after IOP reduction. Therefore, preoperative use of topical medication appears to not have a significant influence on changes in ONH blood flow after surgery [15, 27]. Fourth, we measured LSFG twice after treatment. To strengthen data accuracy, more than three LSFG measurements may be required. Finally, our study sample size was relatively small. Larger studies with repeated LSFG measurements are needed in the future to confirm these findings.

## 5. Conclusion

In conclusion, the findings of the present study suggest that both PG analog instillation and trabeculectomy can change the pulse waveforms for ONH microcirculation. As impaired ocular blood flow plays a role in the progression of glaucomatous damage, it would be beneficial if the treatment for glaucoma could improve the stability of ONH microcirculation.

## Figures and Tables

**Table 1:** Demographic and ocular characteristics of patients with glaucoma treated with prostaglandin analogs or trabeculectomy.

	PG analogs group	Trabeculectomy group
	*N* = 28	*N* = 23
*Demographic characteristics*		
Sex (male/female)	10/18	10/13
Age (years)	50.6 ± 10.8	60.0 ± 9.7

*Ocular characteristics*		
POAG/NTG	4/24	19/4
Refraction (diopter)	−4.3 ± 3.2	−4.3 ± 2.9
MD (dB)	−4.8 ± 5.0	−14.3 ± 7.4
PSD (dB)	7.6 ± 4.0	11.8 ± 4.1
CCT (*μ*m)	520.9 ± 30.5	520.8 ± 28.2
Pretreatment IOP (mmHg)	16.0 ± 2.8	19.1 ± 3.8
Pretreatment MOPP (mmHg)	43.0 ± 12.5	43.0 ± 11.7
Pretreatment heart rate (beats/min)	70.9 ± 12.6	66.7 ± 11.9
Pretreatment MBP (mmHg)	85.6 ± 13.2	93.2 ± 15.7
Intraocular pressure decrease rate (%)	26.0 ± 5.0	42.2 ± 14.3

*Clinical characteristics*		
Hypertension	4	6
Pretreatment glaucoma medications	0	23
Prostaglandin analogs	0	23 (100%)
Beta-blockers	0	20 (87.0%)
Carbonic anhydrase	0	22 (95.7%)
Rho kinase inhibitors	0	9 (39.1%)
Alpha-2-sympathomimetic drugs	0	18 (78.3%)

The data are presented as means ± standard deviations. POAG: primary open-angle glaucoma; NTG: normal-tension glaucoma; MD: mean deviation; PSD: pattern standard deviation; CCT: central cornea thickness; IOP: intraocular pressure; MOPP: mean ocular perfusion pressure; MBP: mean blood pressure; PG: prostaglandin analogs.

**Table 2:** Changes in ocular and systemic parameters at 3 months after the instillation of topical prostaglandin analogs (*N* = 28).

	Before treatment	After treatment	*p* value^*∗*^
IOP (mmHg)	15.95 ± 2.81	11.82 ± 2.31	0.001
MOPP (mmHg)	42.95 ± 12.48	46.25 ± 7.18	0.004
MBP (mmHg)	85.58 ± 13.15	87.70 ± 11.73	0.175
Heart rate (beats/min)	70.92 ± 12.57	69.90 ± 10.41	0.657
MBR_T_ (AU)	10.43 ± 1.65	10.10 ± 1.80 (97.2 ± 11.0)	0.136 (0.143)
Fluctuation (AU)	13.05 ± 3.44	12.23 ± 3.34 (94.8 ± 15.7)	0.106 (0.122)
BOS (AU)	77.39 ± 5.79	78.65 ± 5.89 (101.7 ± 4.8)	0.122 (0.116)
ATI (AU)	31.56 ± 3.58	30.44 ± 3.82 (96.7 ± 7.8)	0.034 (0.031)
BOT (AU)	51.81 ± 4.72	51.89 ± 4.75 (99.8 ± 6.2)	0.909 (0.982)
Skew (AU)	11.13 ± 1.94	11.34 ± 1.90 (102.9 ± 13.4)	0.301 (0.290)
Number of glaucoma medications	0	1	<0.001

The data are presented as means ± standard deviations. ^*∗*^Differences in values before and after treatment were determined using analysis of variance with the Wilcoxon test. The ratios calculated based on the pretreatment values and *p* values based on the ratios were presented in the square brackets. IOP: intraocular pressure; MOPP: mean ocular perfusion pressure; MBP: mean blood pressure; MBR_T_: mean blur rate in the tissue area of the optic disc; AU: arbitrary unit; BOS: blowout score; ATI: acceleration time index; BOT: blowout time.

**Table 3:** Changes in ocular and systemic parameters at 3 months after trabeculectomy for glaucoma (*N* = 23).

	Before treatment	After treatment	*p* value^*∗*^
IOP (mmHg)	19.1 ± 3.8	10.8 ± 2.9	<0.001
MOPP (mmHg)	43.0 ± 11.7	51.4 ± 8.6	0.002
MBP (mmHg)	93.2 ± 15.7	93.8 ± 15.1	0.843
Heart rate (beats/min)	66.7 ± 11.9	65.2 ± 6.7	0.721
MBR_T_ (AU)	8.0 ± 2.1	7.8 ± 2.2 (98.6 ± 16.9)	0.301 (0.412)
Fluctuation (AU)	13.3 ± 3.5	12.1 ± 3.3 (92.4 ± 19.3)	0.019 (0.036)
BOS (AU)	76.3 ± 5.9	78.5 ± 5.4 (103.1 ± 6.5)	0.036 (0.033)
ATI (AU)	32.9 ± 4.2	32.4 ± 5.3 (99.3 ± 17.1)	0.503 (0.761)
BOT (AU)	50.8 ± 5.3	50.8 ± 5.3 (100.5 ± 9.0)	0.939 (0.976)
Skew (AU)	10.3 ± 2.2	10.8 ± 2.4 (107.1 ± 22.9)	0.162 (0.094)
Number of glaucoma medications	3.3 ± 0.9	0	<0.001

The data are presented as means ± standard deviations. ^*∗*^Differences in values before and after eye treatment were determined using analysis of variance with the Wilcoxon test. The ratios calculated based on the pretreatment values and *p* values based on the ratios were presented in the square brackets. IOP: intraocular pressure; MOPP: mean ocular perfusion pressure; MBP: mean blood pressure; MBR_T_: mean blur rate in the issue area of the optic disc; AU: arbitrary unit; BOS: blowout score; ATI: acceleration time index; BOT: blowout time.

## Data Availability

The data used to support the findings of this study are available from the corresponding author upon request.
